# The Impact of Nurse Staffing and Education on 30‐Day Mortality Among Patients Hospitalized for Acute Kidney Injury

**DOI:** 10.1002/nur.70024

**Published:** 2025-09-29

**Authors:** Christin Iroegbu, Anne Kutney‐Lee, Jesse Chittams, Sheridan Leak, Margo Brooks‐Carthon

**Affiliations:** ^1^ University of Pennsylvania School of Nursing Philadelphia Pennsylvania USA; ^2^ Center for Health Outcomes and Policy Research Philadelphia Pennsylvania USA; ^3^ Leonard Davis Institute for Health Economics Philadelphia Pennsylvania USA; ^4^ Department of Nursing Towson University Towson Maryland USA; ^5^ Veteran Experience Center Corporal Michael J. Crescenz VA Medical Center Philadelphia Pennsylvania USA; ^6^ Center for Health Equity and Research Promotion Corporal Michael J. Crescenz VA Medical Center Philadelphia Pennsylvania USA; ^7^ Saint Joseph's University Philadelphia Pennsylvania USA; ^8^ Children's Hospital of Philadelphia Philadelphia Pennsylvania USA

**Keywords:** acute kidney failure, acute renal failure, hospital mortality, nurse staffing, nursing, nursing education

## Abstract

Acute kidney injury (AKI) affects approximately 20% of hospitalized patients and is associated with higher mortality, extended hospital stay, and increased costs. While various strategies have been proposed to improve AKI management, the impact of nursing resources on AKI outcomes has not been explored. We sought to examine the association between nursing resources and 30‐day mortality among patients hospitalized with AKI. Using a cross‐sectional study design, we linked data from the CMS Medicare Provider Analysis and Review file, American Hospital Association Annual Survey, and RN4CAST‐NY/IL survey of registered nurses. We identified 24,368 Medicare beneficiaries aged 18–99 years with a primary diagnosis of AKI hospitalized in 155 hospitals in New York and Illinois in 2021. The primary outcome was 30‐day mortality. Key independent variables included nurse staffing (patient‐to‐nurse ratio) and nurse education (proportion of nurses holding a bachelor's degree or higher). Covariates were patient demographics, comorbidities, and hospital characteristics. The 30‐day mortality rate was 10.5%. In adjusted logistic regression models, each additional patient per RN increased the odds of 30‐day mortality by 7% (OR = 1.07, 95% CI [1.01–1.13], *p* < 0.05). For each 10‐point increase in the proportion of nurses with a bachelor's degree or higher, the odds of 30‐day mortality decreased by 9% (OR = 0.91, 95% CI [0.88–0.95], *p* < 0.001). Better nurse staffing and higher proportions of nurses with a bachelor's degree or higher are associated with lower 30‐day mortality among patients hospitalized with AKI. These findings underscore the significance of nursing in AKI outcomes and suggest that hospitals should prioritize investing in nursing resources to enhance AKI outcomes.

Acute kidney injury (AKI) is a severe condition affecting approximately 20% of hospitalized patients and is characterized by a rapid decline in kidney function (Yousef Almulhim 2025). Patients with AKI face substantially prolonged hospital stays, and a higher likelihood of developing chronic kidney disease and risk of in‐hospital mortality (Aklilu et al. 2024; Schulman et al. 2023). The significant burden and increased costs of AKI management and its sequelae on healthcare systems have led to substantial public health concerns (Ostermann and Joannidis 2016; Schulman et al. 2023). The societal burden that ensues from the long‐term effects of AKI has prompted global health campaigns that highlight that AKI is preventable, its course is modifiable, and its treatment with high‐quality care can improve patient outcomes (Fortin and Boucher 2021; Schulman et al. 2023).

Approaches to mitigate the adverse consequences of AKI have been developed, including the investigation of novel biomarkers for early AKI detection, educational interventions for healthcare professionals, and hospital electronic alert systems (Wilson et al. 2021; Selby et al. 2016; Yousef Almulhim 2025). Still, the routine use and effectiveness of these strategies in clinical settings remain limited, underscoring a continued need for research on alternative methods to enhance AKI management and outcomes (Ostermann and Joannidis 2016; Vijayan 2021; White et al. 2013). The gold standard for AKI prevention and treatment remains the optimization of hemodynamic and volume status and surveillance for clinical deterioration, both of which are critical functions of the registered nurse (Al‐ghraiybah et al. 2024; Schulman et al. 2023; Vijayan 2021).

Surveillance, or the ability of nurses to monitor, evaluate, and act upon changes in a patient's clinical status, is heavily influenced by organizational features, including nurse staffing and educational preparation (Halverson and Scott Tilley 2022; Kim and Cho 2023). Several studies have demonstrated a clear association between nurse staffing levels, nurse education, and patient mortality rates (Dall'Ora et al. 2022). For example, higher nurse workload has been linked to increased 30‐day mortality following surgery, and similar associations have been reported with myocardial infarction and sepsis (Brooks Carthon et al. 2021; Dierkes et al. 2022; Lasater et al. 2021). Nurses with a baccalaureate degree or higher are also associated with improved patient outcomes, likely due to advanced training in critical thinking, patient safety, and evidence‐based practice in acute care hospitals (Buxton and Wang 2023; Djukic et al. 2019; Porat‐Dahlerbruch et al. 2022). In contrast, associate degree or diploma programs focus primarily on technical nursing skills, but not on the broader educational breadth of BSN programs. A systematic review found consistent associations between higher proportions of nurses holding a baccalaureate of science degree in nursing (BSN) and lower mortality risk in a wide range of hospitalized patient populations (Audet et al. 2018). These findings suggest that highly educated nurses may be better equipped to identify and respond to complex care situations, highlighting the critical role that advanced nursing education plays in enhancing patient safety, reducing mortality, and improving the delivery of high‐quality care (Al‐ghraiybah et al. 2024).

Despite this robust evidence, few studies have specifically examined how these nursing resources relate to outcomes in patients hospitalized with AKI, a population at particularly high risk for adverse events and for whom timely, high‐quality nursing care may be especially consequential. Most existing research in AKI has been conducted outside of the United States and has focused on nurses’ knowledge or risk identification, rather than directly linking nurse staffing and education to patient outcomes (Ellis 2022; Fortin and Boucher 2021; Parker and Chu 2024; K. C. Wang and Moore 2023). Therefore, our study addresses this gap by investigating the association between nursing resources (i.e., nurse staffing and nurse education) and 30‐day mortality among patients hospitalized with AKI. In doing so, we extend the established literature on nursing and patient outcomes to a clinically significant and vulnerable subgroup, providing evidence to inform targeted strategies for improving care and outcomes in this high‐risk population.

## 1 Materials and Methods

### 1.1 Study Design

This cross‐sectional observational study linked data using a common hospital identifier from three sources: the Center for Medicare and Medicaid (CMS) Medicare Provider Analysis and Review (MedPAR) file, the American Hospital Association (AHA) Annual Survey, and the RN4CAST‐NY/IL survey of registered nurses (RNs), in New York and Illinois, collected between April and June 2021. The 2021 CMS MedPAR file provided patient demographic and clinical information for hospitalized Medicare beneficiaries with a primary diagnosis of AKI. The 2021 AHA Annual Survey provided data on the hospital characteristics, including bed size, teaching status, and technology capabilities. New York and Illinois were selected for this survey because both states were actively considering hospital nurse staffing legislation at the time of data collection, and there was a critical need for timely, local evidence to inform these policy debates. The two states encompass a diverse array of hospital types and patient populations, exhibiting considerable variation in nurse staffing practices. While findings are most directly applicable to NY and IL, the diversity within these states enhances the relevance of our results to other regions considering similar policy interventions (Lasater et al. 2021).

#### 1.1.1 Nurse Survey Instrument

The RN4CAST‐NY/IL survey provided information about nurse staffing and nurse education levels within hospitals. Nurses were asked to provide the name of their primary place of employment, allowing responses to be aggregated by specific hospital. The National Council of State Boards of Nursing emailed surveys to all actively licensed RNs in New York and Illinois. This direct approach via licensing registries was chosen to avoid potential nonresponse bias from hospitals that may be less inclined to participate if they have poorer nursing resources and a less supportive culture. This approach also enabled us to collect consistent data on organizational nursing characteristics when conducting a study across hundreds of hospitals in New York and Illinois (Aiken et al. 2014). Non‐respondents received a follow‐up email invitation to complete the survey over 2 months using the Dillman method (Lasater et al. 2019). Previous studies using this double‐sampling approach of non‐respondents found no significant differences between respondents and non‐respondents. This suggests that nonresponse bias was likely minimal (Lasater et al. 2019).

The RN4CAST‐NY/IL survey achieved an 18% response rate from over half a million licensed RNs, which is typical for recent electronic surveys (Aiken et al. 2023). On average, each study hospital had 47 nurse respondents. The hospital sample comprised 60% of general acute care hospitals in New York and Illinois. Of these, 94% were hospitals with more than 100 beds, which collectively cared for the majority of patients in this study. Respondents provided information about their employment setting (e.g., hospital, nursing home) and position (e.g., staff nurse, nurse manager). In this study, we included direct care staff nurses in acute care hospitals and excluded RNs not involved in adult inpatient care. Of the 24,114 responses from hospital‐based RNs in direct care, only those from hospitals with at least 10 respondents were used to create reliable hospital‐level estimates of the nursing resources (Lasater et al. 2019). This aggregation methodology has been previously described and has been used for over two decades to study the associations between organizational nursing factors and patient outcomes in the U.S. and internationally (Aiken et al. 2002, 2023; Lasater et al. 2019; Sloane et al. 2018).

#### 1.1.2 Sample

Our final sample included 155 acute care and general hospitals with at least 10 RN4CAST‐NY/IL survey respondents, had AKI admissions in 2021, were in New York or Illinois, and participated in the AHA Annual Survey. Our patient sample consisted of 24,368 Medicare beneficiaries aged 18–99 years who were hospitalized between January 1 and December 31, 2021. These patients had a primary diagnosis of AKI, identified by the International Classification of Diseases, 10th Revision (ICD‐10) codes N17.9, N17.1, N17.0, N17.2, N17.8, T79.5XXA, and N99.0. To identify the main effect of AKI on 30‐day mortality, we excluded individuals with a history of end‐stage renal disease (identified through the ICD‐10 code N18.6) (Wang et al. 2012). We also excluded patients with a length of stay of less than 24 h.

### 1.2 Measurement

Our outcome of interest was 30‐day mortality, defined as death occurring during admission or outside of the hospital within 30 days of admission. Key independent variables of interest derived from the nurse survey included nurse staffing (measured as the average patient‐to‐RN ratio in the hospital) and nurse education (measured as the proportion of BSN‐prepared RNs in the hospital).

#### 1.2.1 Nurse Staffing

Hospital‐level staffing ratios were derived from direct care nurses who reported working in medical or surgical units (including oncology). On the nurse survey, direct care RNs provided information on the number of patients and RNs on the unit during their last shift. To calculate a patient‐to‐RN ratio for individual nurses, we divided the number of patients by the number of nurses working on the unit during the last shift (Brooks Carthon et al. 2021). To create a hospital‐level nurse staffing measure, we calculated the average patient‐to‐RN ratio among nurses working on medical‐surgical units within the same hospital. This staffing measure offers advantages over alternative methods that often incorporate nurses in non‐direct care roles, leading to an underestimation of the workload of direct care nurses. On the nurse survey, nurses were asked to report their highest level of education completed in nursing (i.e., diploma, associate degree, baccalaureate degree, master's degree, doctoral degree). Our measure of nursing education was calculated as the proportion of RNs holding a BSN degree or higher in each hospital.

#### 1.2.2 Patient and Hospital Characteristics

Patient characteristics obtained from the MedPAR file included age (categorized as younger [18–65 years] vs. older [≥ 65 years]), sex as a biological variable (male/female), race/ethnicity (White, Black, Hispanic, Asian, Other), and comorbidities commonly associated with AKI mortality (heart failure, diabetes mellitus, hypertension, and liver disease) (Malbrain et al. 2024). Hospital characteristics obtained from the AHA Annual Survey included bed size, technology status, teaching status, and location. Hospital size was classified as small (≤ 100 beds), medium (101–250 beds), or large (> more than 250 beds). Teaching status was measured by calculating the ratio of medical residents and fellows to beds, with nonteaching hospitals having no residents/fellows, minor teaching hospitals having 0–4 residents/fellows per bed, and major teaching hospitals having more than four residents/fellows per bed. Minor and major teaching categories were combined to create a binary variable (nonteaching vs. teaching) for analysis. High‐technology status was identified as a hospital with the capability to perform open‐heart surgery and/or major organ transplants. To categorize hospital location, we used the Core‐Based Statistical Area (CBSA), a U.S. Census‐based measure of population density. Metropolitan areas were classified as regions with 50,000 or more residents, micropolitan areas with 10,000–49,999 residents, and rural areas with fewer than 10,000 residents. We combined rural and micropolitan due to the small number of hospitals in rural areas. We also accounted for the specific states, New York (NY) and Illinois (IL), where each hospital was located.

### 1.3 Methods

Descriptive statistics were used to describe the characteristics of AKI patients and hospitals included in the study. Unadjusted and adjusted logistic regression models were used to assess the independent effect of nurse staffing and nurse education on 30‐day mortality. Adjusted models included a set of patient (age, race, sex, comorbidities, hypertension, diabetes, heart failure, and liver disease) and hospital (teaching status, technology status, size, urban/rural location, and state) covariates. We adjusted to account for the clustering of patients within hospitals (Freedman 2006). To interpret the effect of nurse education, the percentage of BSN‐prepared nurses was rescaled so that a one‐unit change equaled a 10‐percentage point change. The analysis yielded odds ratios from models that jointly estimated the association between each nursing resource (patient‐to‐RN ratio and proportion of BSN‐prepared nurses) and 30‐day mortality.

Model performance and assumption tenability were evaluated using the C‐statistic to differentiate between those with and without the outcome, Hosmer‐Lemeshow test for overall goodness of fit with 20 groups (nonsignificant *p*‐value > 0.05 indicating adequate fit), and variance inflation factors (VIFs) for multicollinearity (values < 5.0 acceptable). Statistical significance was set for a p‐value of 0.05. Analyses were conducted using STATA 18.0. The EQUATOR STROBE guidelines were used for reporting.

## 2 Results

The demographic and clinical characteristics of the sample are summarized in Table 1. A total of 24,368 patients were admitted with a primary diagnosis of AKI. Older adults comprised over half of the patient sample, with 21,945 (90.1%) older than 65. There were approximately equal numbers of males and females, with 12,041 (49.4%) male patients and 12,327 (50.6%) female patients. The patient sample was predominantly White (*n* = 16,816, 69.0%), followed by Black (*n* = 5196, 21.3%), Hispanic (*n* = 868, 3.6%), and Asian (*n* = 537, 2.2%). A total of 951 (3.9%) patients had an unidentified race. Hypertension was the most common comorbidity, affecting 88.6% (*n* = 21,592) of the patients. Over one‐third of the sample (*n* = 9206, 37.8%) had a diagnosis of heart failure, and nearly half (*n* = 11,894, 48.8%) had diabetes mellitus. Of the included comorbidities, liver failure was the least common and was present in 7.1% (*n* = 1732) of patients. A total of 2560 (10.5%) patients admitted with a primary diagnosis of AKI died within 30 days of admission.

**TABLE 1:** Mortality outcomes and patient characteristics for hospitalized AKI patients (*N* = 24,368).

Patient Characteristics	
Age	
Younger (18–64)	2423 (9.9%)
Older (≥ 65)	21,945 (90.1%)
Sex	
Male	12,041 (49.4%)
Female	12,327 (50.6%)
Race	
White	16,816 (69.0%)
Black	5196 (21.3%)
Hispanic	868 (3.6%)
Asian	537 (2.2%)
Other	951 (3.9%)
Comorbidities	
Heart Failure	9206 (37.8%)
Diabetes Mellitus	11,894 (48.8%)
Hypertension	21,592 (88.6%)
Liver Failure	1732 (7.1%)
30‐day mortality	2560 (10.5%)

Table 2 provides characteristics of the 155 hospitals included in this study. The average staffing ratio on an adult medical‐surgical unit was 5.4 patients per nurse, SD = 1.00 (range: 1.75–9.43). In the average study hospital, 75% of nurses held a BSN degree or higher, SD = 18% (range: 18%–100%). Sixty‐six hospitals (42.6%) were located in Illinois, and 89 hospitals (57.4%) were located in New York. Most hospitals were in metropolitan regions (91.6%). Most hospitals in the sample were extensive facilities with over 250 beds (60.7%), had high technology status (51.6%), and were either major teaching hospitals (31.6%) or nonteaching hospitals (41.3%).

**TABLE 2:** Hospital characteristics (*n* = 155 hospitals).

Proportion of nurses with a BSN degree or higher, mean (SD)	0.75 (0.18)
Medical‐Surgical Staffing, mean (SD)	5.4 (1.00)
Medical‐Surgical Staffing, n (%)	
< 5 patient/nurse	56 (36.1%)
5–7 patient/nurse	92 (59.4%)
> 7 patients/nurse	7 (4.5%)
Beds, n (%)	
< 100	9 (5.8%)
101–250	52 (33.6%)
> 250	94 (60.7%)
High Technology, n (%)	80 (51.6%)
Teaching Status, n (%)	
None	64 (41.3%)
Minor	42 (27.1%)
Major	49 (31.6%)
Location, n (%)	
Metro	142 (91.6%)
Rural	13 (8.4%)
State, n (%)	
Illinois	66 (42.6%)
New York	89 (57.4%)

Table 3 displays the unadjusted and adjusted odds ratios indicating the effects of each nursing resource on the odds of 30‐day mortality among hospitalized patients with AKI. In the unadjusted model, the relationship between the patient‐per‐nurse ratio and 30‐day mortality was not significant (OR, 1.05; 95% CI [0.99–1.11]; *p* = 0.07). However, after controlling for patient and hospital characteristics, a statistically significant relationship was observed between the patient‐to‐RN ratio and 30‐day mortality, with the odds of mortality being 7% higher (OR, 1.07; 95% CI [1.01–1.13]; *p* < 0.05) for each additional patient‐per‐RN. The relationship between the proportion of BSN‐prepared nurses and 30‐day mortality was significant in both the unadjusted and adjusted models. Both unadjusted and adjusted analyses showed that higher proportions of BSN‐prepared nurses were significantly associated with reduced 30‐day mortality risk. Specifically, each 10% point increase in the proportion of RNs with a BSN or higher degree was associated with a 9% reduction in the odds of 30‐day mortality (unadjusted OR 0.91, 95% CI [0.88–0.94], *p* < 0.001; adjusted OR 0.91, 95% CI [0.88–0.95], *p* < 0.001). This association remained consistent even after controlling for patient and hospital characteristics.

**TABLE 3:** Effects of nursing resources on odds of 30‐day mortality among hospitalized AKI patients (*n* = 24,368).

Unadjusted					Adjusted			
	*B*	SE	*z*	Odds Ratio (95% CI)	*B*	SE	*z*	Odds Ratio (95% CI)
Predictor								
Each additional patient‐per‐RN (Med/Surg staffing ratio)	0.050	0.029	1.79	1.05 (0.99–1.11)	0.07	0.030	2.36	1.07 (1.01–1.13)*
10‐point increase in proportion of RNs with a BSN or higher	−0.093	0.015	−5.74	0.91 (0.88–0.94)**	−0.09	0.017	−4.58	0.91 (0.88–0.95)**
Age								
Older adult (≥ 65)		—			0.96	0.31	8.18	2.62 (2.08–3.29)**
Race								
Black		—			−0.33	0.04	−5.41	0.71 (0.63–0.81)**
Hispanic		—			−0.48	0.10	−3.07	0.62 (0.45–0.84)**
Asian		—			0.03	0.21	0.12	1.03 (0.69–1.53)
Other		—			−0.27	0.08	−2.59	0.76 (0.62–0.94)*
Gender								
Male		—			0.04	0.04	0.90	1.04 (0.96–1.13)
Comorbidity								
Heart failure		—			0.49	0.77	10.39	1.64 (1.49–1.79)**
Diabetes		—			−0.40	0.03	−8.35	0.67 (0.61–0.74)**
Hypertension		—			−0.36	0.05	−4.68	0.70 (0.60–0.81)**
Liver failure		—			0.45	0.13	5.45	1.57 (1.34–1.85)**
Hospital Characteristics								
Teaching Hospital		—			−0.00	0.07	−0.00	0.99 (0.88–1.14)
High technology		—			0.06	0.07	0.87	1.06 (0.93–1.22)
< 100 beds		—			−0.27	0.14	−1.42	0.76 (0.52–1.11)
101–250 beds		—			0.13	0.08	1.69	1.14 (0.98–1.31)
Rural location		—			−0.22	0.09	−1.88	0.81 (0.64–1.00)
Model Fit								
C‐statistic				0.5462	0.6334			
Hosmer‐Lemeshow χ^2^(18)					28.16, *p* = 0.06			
Mean VIF (range)					1.13 (1.01–1.34)			

*Note:* Odds ratios are from robust logistic regression models that estimate the joint effects of the nursing resources. Adjusted models included patient characteristics (age, race, sex, comorbidities) and hospital characteristics (size, technology status, teaching status, urban/rural location, state). Model performance: C‐statistic; Hosmer–Lemeshow test; mean VIF.

* *p* < 0.05.

** *p* < 0.001.

The logistic regression model demonstrated good ability to differentiate participants who died within 30 days from those who survived (C‐statistic = 0.63, indicating moderate discrimination). The model fit was adequate (Hosmer–Lemeshow χ²(18) = 28.16, *p* = 0.16). All predictors had VIF values close to 1 (range: 1.01‐1.34; mean VIF = 1.13), indicating no multicollinearity concerns.

**FIGURE 1:**
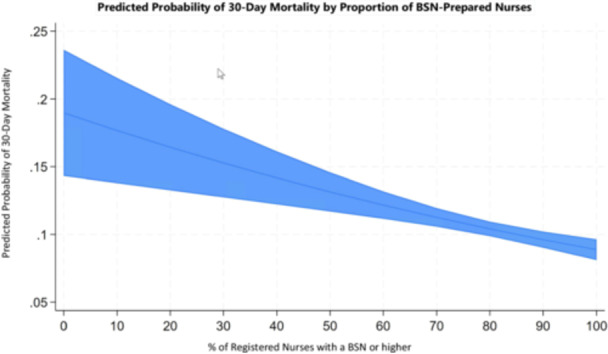
Predicted lives saved per 100 patients by percentage of BSN‐prepared nurses.

We also calculated the number needed to treat (NNT)—in this context, the additional number of BSN‐prepared RNs required to prevent one case of 30‐day mortality. Based on our predictive model, for every 10 out of 100 registered nurses who hold a BSN, we estimate that approximately 9.9 additional lives are saved per 100 patients treated. Because the proportion of BSN‐prepared nurses is measured continuously, even small increases in this percentage lead to corresponding changes in the predicted probability of death. We therefore created a plot (Figure 1) to visually represent the relationship between BSN staffing levels and patient mortality. The graph illustrates that the largest improvement in lives saved occurs when the percentage of BSN‐prepared nurses increases from 0% to 10%. Additional increases in BSN‐prepared staff continue to result in lives saved, though the rate of improvement becomes less pronounced as the proportion approaches 100%.

## 3 Discussion

This study represents one of the first to provide empirical evidence demonstrating an association between hospital nursing resources, including nurse staffing and education levels, and 30‐day mortality among patients hospitalized with AKI. We build on and extend the existing literature by examining how key nursing organizational features are associated with AKI mortality outcomes in acute care settings. In our cross‐sectional analysis of 24,368 patients admitted with a primary diagnosis of AKI, we found that patients cared for in hospitals with lower patient‐to‐RN staffing ratios and higher proportions of nurses with at least a BSN degree had significantly lower odds of 30‐day mortality.

Previous research demonstrates that early recognition and quick intervention are vital for improving outcomes in hospitalized patients with AKI (Aklilu et al. 2024; Wu et al. 2023). As the primary surveillance system in hospitals, nurses play a central role in monitoring, detecting, and managing the clinical consequences of AKI (Al‐ghraiybah et al. 2024; Malbrain et al. 2024). This responsibility requires significant attention to vital signs, urine output, lab results, medication administration, and fluid balance (Ellis 2022). However, maintaining this level of vigilance is especially challenging when nurses care for multiple patients, which can increase the risk of missing critical nursing interventions (Al‐ghraiybah et al. 2024). In these complex and demanding environments, the advanced critical thinking, evidence‐based practice, and leadership skills developed through BSN programs become highly valuable, as they enable nurses make more effective clinical judgments, advocate for patients, and collaborate with interdisciplinary teams (Buxton and Wang 2023; Djukic et al. 2019). These skills are crucial for managing complex conditions, such as AKI, where timely recognition of deterioration and immediate action can significantly impact patient trajectory. Previous research indicates that early recognition and prompt intervention are essential for improving outcomes in hospitalized patients with AKI (Aklilu et al. 2024; Wu et al. 2023). As the main surveillance system in hospitals, nurses play a central role in monitoring, detecting, and managing the clinical consequences of AKI (Al‐ghraiybah et al. 2024; Malbrain et al. 2024). This responsibility requires significant attention to vital signs, urine output, lab results, medication administration, and fluid balance (Ellis 2022). However, maintaining this level of vigilance is especially difficult when nurses care for multiple patients, which can increase the risk of missing essential nursing interventions (Al‐ghraiybah et al. 2024). In these complex and demanding environments, the advanced critical thinking, evidence‐based practice, and leadership skills gained through BSN programs become especially valuable, as they help nurses make better clinical judgments, advocate for patients, and collaborate with interdisciplinary teams (Buxton and Wang 2023; Djukic et al. 2019). These skills are essential for managing complex conditions like AKI, where early detection of deterioration and immediate action can greatly affect outcomes.

Our findings suggest that insufficient nurse staffing and a lower proportion of nurses with a BSN degree or higher are associated with adverse consequences for patients hospitalized with AKI. Improving nurse‐to‐patient staffing ratios and increasing the proportion of BSN‐prepared nurses are system‐level strategies strongly supported by legislative trends, professional recommendations, and national initiatives. Several states have enacted staffing ratio laws, and federal legislation is under consideration to establish national and minimum nurse‐to‐patient ratios (Krishnamurthy et al. 2024). The Future of Nursing Report set a goal for 80% of nurses to hold a BSN, and our analysis of 155 hospitals shows substantial progress, with an average of 75% of nurses being BSN‐prepared; however, further advancement is still needed.

Participation in accredited excellence programs, such as Magnet or Pathways to Excellence, further emphasized the importance of nursing education and adequate nurse staffing (Abuzied et al. 2022; Lal 2024). These programs provide a roadmap for hospital and healthcare organizations to enhance nursing resources, which is associated with improved patient outcomes and overall hospital performance, particularly for patients with AKI, who require timely and high‐quality nursing care. These initiatives underscore the crucial role of nurse education and staffing in patient outcomes, a conclusion supported by our study's findings. Implementing and evaluating such interventions may offer practical, evidence‐based strategies for improving AKI outcomes across healthcare settings.

There are also implications for public policy and professional education in low‐ and middle‐income countries. The World Health Organization advocates for investment in nursing education and workforce expansion in low‐ and middle‐income countries (LMICs) (Azad et al. 2020). Although some LMICs have introduced BSN or equivalent degree programs to align with international standards, diploma and certificate‐level training remains predominant, and BSN programs are less prevalent than in high‐income settings (Jeon et al. 2024; World Health Organization 2020). Findings from this study indicate that increasing the proportion of BSN‐prepared registered nurses is significantly associated with a reduction in mortality among patients with acute kidney injury. This evidence underscores the importance of public policy initiatives that standardize nursing education and enhance workforce development in LMICs, providing a critical and sustainable pathway to improved health system performance and enhanced patient outcomes.

These results underscore the importance of considering nursing resources in future research on outcomes related to AKI care. While the complexity of AKI management is notable, it is essential to acknowledge that most hospitalized patients today present with complex conditions that require vigilant surveillance and skilled clinical judgment, supported by adequate nurse staffing and higher levels of nursing education. Additionally, the approach of surveying nurses and estimating the relationships between education and patient outcomes has been used in prior studies with larger samples of nurses, across more states, and internationally. This suggests that the model employed in this study can be feasibly replicated, thereby enhancing the generalizability of the findings to broader contexts. Our study contributes to the growing body of evidence demonstrating that a well‐educated and adequately resourced nursing workforce is essential for the management of AKI and the broader spectrum of complex inpatient care. By reinforcing the critical impact of nurse staffing and education on patient outcomes, these findings support ongoing efforts to inform nursing practice, guide professional development, and shape policy to optimize care for all hospitalized patients with complex needs.

### 3.1 Limitations

There are some limitations to this study that affect the interpretation of our findings. The cross‐sectional design of this study does not permit the establishment of causal relationships between nursing resources and patient outcomes; instead, it identifies associations that should be interpreted with caution. Additionally, we acknowledge that generalizability may be limited in settings with substantially different healthcare infrastructures or regulatory contexts, and the results from this study may not be fully applicable to other regional or types of hospitals with distinct patient populations, staffing models, or healthcare systems. The nurse survey data were aggregated at the hospital level and may not accurately reflect the specific units where patients received care. However, AKI patients may be cared for across multiple units during hospitalization, including general care areas and intensive care units. Therefore, the hospital‐level assessment may be a more general representation of their exposure to nursing. Lab values (e.g., serum creatinine, blood urea nitrogen, estimated glomerular filtration rate) indicating the severity of AKI could not be retrieved from the MedPAR data file, which may have provided the most accurate marker of renal impairment had it been available (Malbrain et al. 2024). However, we leveraged prior research demonstrating the high specificity (97.7%) and low sensitivity (35.4%) of ICD‐9‐CM codes in identifying cases of AKI for this analysis (Mittalhenkle et al. 2008; Waikar et al. 2006). Finally, although we adjusted for four comorbidities most associated with AKI mortality, we did not have measures for all potential confounders that may have influenced mortality, such as baseline kidney function, severity of AKI, or other metabolic complications (Abebe et al. 2021; Gameiro et al. 2020).

## 4 Conclusion

Our findings of a significant association between lower mortality, a higher proportion of BSN‐prepared nurses in hospitals, and lower patient‐to‐RN ratios among hospitalized patients with AKI add a novel contribution to the nephrology nursing literature. Our findings also support efforts for safe nurse staffing policies and recommendations for hospitals having 80% of nurses with a BSN degree, which have significant implications for nursing practice, nurses’ ability to deliver high‐quality care, and improving outcomes for patients experiencing AKI. Importantly, these findings underscore the need for ongoing institutional investment in the academic advancement of the nursing workforce. Policymakers and nursing leaders are encouraged to leverage these findings to strengthen and sustain advocacy for educational initiatives that increase the proportion of BSN‐prepared nurses, including tuition reimbursement for associate degree Nurses and university RN‐to‐BSN programs. Continued investment in academic advancement is essential for maintaining high standards of nursing care and achieving improved patient outcomes.

## Author Contributions

**Christin Iroegbu:** conceptualization, investigation, data curation, data analysis, visualization, writing – original draft preparation, editing. **Anne Kutney‐Lee:** conceptualization, methodology, writing – review and editing, supervision. **Jesse Chittams:** data curation, data analysis, methodology, review and editing. **Sheridan Leak:** data curation, writing – original draft preparation, visualization. **Margo Brooks‐Carthon:** conceptualization, methodology, writing – review and editing, supervision.

## Consent

Registered nurses from New York and Illinois completed an electronic survey for this study.

## Conflicts of Interest

The authors declare no conflicts of interest.

## Data Availability

The data that support the findings of this study are available on request from the corresponding author. The data are not publicly available due to privacy or ethical restrictions.
